# Identification and characterization of long non-coding RNAs involved in osmotic and salt stress in *Medicago truncatula* using genome-wide high-throughput sequencing

**DOI:** 10.1186/s12870-015-0530-5

**Published:** 2015-06-06

**Authors:** Tian-Zuo Wang, Min Liu, Min-Gui Zhao, Rujin Chen, Wen-Hao Zhang

**Affiliations:** State Key Laboratory of Vegetation and Environmental Change, Institute of Botany, the Chinese Academy of Sciences, Beijing, 100093 People’s Republic of China; Research Network of Global Change Biology, Beijing Institutes of Life Science, the Chinese Academy of Sciences, Beijing, 100101 People’s Republic of China; Plant Biology Division, The Samuel Roberts Noble Foundation, Ardmore, OK 73401 USA

**Keywords:** Long non-coding RNAs (lncRNAs), Osmotic stress, Salt stress, *Medicago truncatula*, Legume plants, High-throughput sequencing, Transcriptional regulation

## Abstract

**Background:**

Long non-coding RNAs (lncRNAs) have been shown to play crucially regulatory roles in diverse biological processes involving complex mechanisms. However, information regarding the number, sequences, characteristics and potential functions of lncRNAs in plants is so far overly limited.

**Results:**

Using high-throughput sequencing and bioinformatics analysis, we identified a total of 23,324 putative lncRNAs from control, osmotic stress- and salt stress-treated leaf and root samples of *Medicago truncatula*, a model legume species. Out of these lncRNAs, 7,863 and 5,561 lncRNAs were identified from osmotic stress-treated leaf and root samples, respectively. While, 7,361 and 7,874 lncRNAs were identified from salt stress-treated leaf and root samples, respectively. To reveal their potential functions, we analyzed Gene Ontology (GO) terms of genes that overlap with or are neighbors of the stress-responsive lncRNAs. Enrichments in GO terms in biological processes such as signal transduction, energy synthesis, molecule metabolism, detoxification, transcription and translation were found.

**Conclusions:**

LncRNAs are likely involved in regulating plant’s responses and adaptation to osmotic and salt stresses in complex regulatory networks with protein-coding genes. These findings are of importance for our understanding of the potential roles of lncRNAs in responses of plants in general and *M. truncatula* in particular to abiotic stresses.

**Electronic supplementary material:**

The online version of this article (doi:10.1186/s12870-015-0530-5) contains supplementary material, which is available to authorized users.

## Background

Non-coding RNAs (ncRNAs) are a set of RNAs that have no capacity to code for proteins. They are used to be considered as inconsequential transcriptional “noises”, because of limited information for their functions [1, 2]. However, this situation is being changed. Recent studies have shown that ncRNAs play important regulatory roles in numerous biological processes [3, 4].

NcRNAs are grouped into small RNAs, such as microRNAs (miRNAs) and small interfering RNAs (siRNAs), and long non-coding RNAs (lncRNAs) according to the length [5]. LncRNAs are defined as a group of ncRNAs that have a length of more than 200 nucleotides [6]. They are usually expressed at low levels and lacking sequence similarities among species, exhibit tissue and cell-specific expression patterns, and transcripts are localized to subcellular compartments [4, 7]. LncRNAs can be further grouped into sense, antisense, bidirectional, intronic and intergenic lncRNAs according to their relative locations with protein-coding genes [8]. In *Arabidopsis thaliana*, >30 % of lncRNAs are intergenic, and antisense lncRNAs are also abundant [9, 10].

It has been shown that some lncRNAs regulate the expression of genes in a close proximity (*cis*-acting) or in a distance (*trans*-acting) in the genome via a number of mechanisms, including modifying promoter activities by nucleosome repositioning, histone modifications, DNA methylation, activating/gathering/transporting of accessory proteins, epigenetic silencing and repression [8, 11, 12]. Increasing evidence supports that lncRNAs play a crucial role in disease occurrence, genomic imprinting and developmental regulation in mammals [13–15].

In contrast to extensive studies of lncRNAs in mammals [13, 14, 16, 17], only a few studies have been reported of the function of lncRNAs in plants [18, 19]. For example, *COOLAIR* and *COLDAIR* have been identified to be associated with *FLOWERING LOCUS C* (*FLC*) in *Arabidopsis. COLDAIR* includes two antisense lncRNAs transcribed from the antisense strand of *FLC*, while *COLDAIR* is an intronic lncRNA transcribed from the first intron of *FLC*. They have been implicated in silencing and epigenetic repression of *FLC* to regulate flowering time [20, 21]. *AtIPS1* and *At4* have been shown to act as target mimics of miR399 by binding and sequestering miR399 and reduce miR399-mediated cleavage of *PHO2* which is important for phosphate uptake [22, 23]. Genome-wide identification of lncRNAs in *A. thaliana* has been reported in several studies [24–27]. In rice, *LDMAR* has been shown to regulate photoperiod-sensitive male sterility [28]. Bioinformatics analyses reveal that 60 % of lncRNAs are precursors of small RNAs and 50 % of lncRNAs are expressed in a tissue-specific manner [29–31].

*Medicago truncatula* is a model legume widely used in genomics, genetics and physiological studies of legumes due to its small genome size and relative ease in genetic transformation [32, 33]. Legumes account for one third of primary crop production in the word and are important sources of dietary proteins for human and animals [34]. In *M. truncatula*, *Enod40* and *Mt4* involved in nodulation and phosphate uptake, respectively, have been identified as lncRNAs [35, 36]. Although a recent *in silico* analysis of lncRNAs has been conducted in *M. truncatula*, only limited information is presented, because only lncRNAs with poly(A) tails have been analyzed, using less finished genome sequences available at the time [37]. As most lncRNAs have no poly(A) tails and are lowly and specifically expressed [4, 16], to identify a comprehensive set of lncRNAs including non-poly(A)-tailed lncRNAs in *M. truncatula*, we conducted genome-wide high-throughput sequencing of six libraries prepared using complementary sequences of synthetic adaptors. Similar to other plant species, legumes are also frequently encountered adverse environments such as osmotic and salt stresses. Previous studies of molecular mechanisms underlying plant’s tolerance to abiotic stresses are mainly focused on functional studies of protein-coding genes, while few studies have systemically investigated the roles of lncRNAs in osmotic and salt stress responses of plants. In the present study, we identified a comprehensive set of lncRNAs that are responsive to osmotic and salt stresses in leaves and roots of *M. truncatula* using high throughput sequencing of six cDNA libraries.

## Results

### Physiological response to osmotic and salt stress

Materials used to construct cDNA libraries were treated by osmotic or salt stress for 5 h. Foliar osmolality was increased from 350 mOsmol kg^−1^ to 450 and 390 mOsmol kg^−1^, after the treatments with osmotic and salt stress, respectively (Table 1). There was a significant increase in foliar Na^+^ concentration after 5-h salt treatment (Table 1). No effects of osmotic and salt stress on concentrations of proline (Pro) and soluble sugars were detected (Table 1). These results suggest that plants under our treatment regime are at the early stage of stress-response to activate genes and their regulatory networks.

**Table 1:** The physiological response of leaves after osmotic or salt stress for 5 h

	Osmolality (mOsmol Kg^−1^)	Na^+^ concentration (mg g^−1^ DW)	Pro concentration (mg g^−1^ DW)	Soluble sugars (mg g^−1^ DW)
Control	350 ± 10.41	0.79 ± 0.09	0.68 ± 0.04	4.83 ± 0.13
Osmotic stress	450 ± 10.58**	0.76 ± 0.12	0.64 ± 0.03	5.09 ± 0.09
Salt stress	390 ± 11.27*	7.26 ± 0.30**	0.66 ± 0.03	4.94 ± 0.08

Data are the means ± SE (*n* = 3). Data with “*” or “**” indicate significant different (*P* < 0.05 or *P* < 0.01) between treatments and control

### High-throughput sequencing

Six cDNA libraries were constructed using mRNA isolated from leaves and roots of *M. truncatula* seedlings treated with osmotic stress (OS), salt stress (SS), and control (CK) and complementary sequences of synthetic adaptors. They were sequenced by an Illumina-Solexa sequencer. The high-throughput sequencing led to more than 90,000,000 raw sequence reads. To assess the quality of RNA-seq data, each base in the reads was assigned a quality score (Q) by a phred-like algorithm using the FastQC [38]. The analysis revealed that the data are highly credible with a mean Q-value of 36 (Additional file 1: Figure S1). Of the raw reads, more than 99 % were clean reads after initial processing (Table 2). We performed 100 bp paired-end sequencing, and led to 56.7 G raw bases and 56.6 G clean bases in total.

**Table 2:** Statistical data of the RNA-Seq reads for six samples

	Control		Osmotic stress		Salt stress	
	Leaf	Root	Leaf	Root	Leaf	Root
Raw reads	96,246,252	94,158,974	95,096,112	92,413,632	91,387,634	92,506,618
Clean reads	95,999,176	93,999,446	94,868,266	92,257,986	91,171,136	92,348,598
Unique lncRNAs	11,501	18,275	8,571	18,277	10,458	19,186
Unique mRNAs	31,034	36,482	29,770	36,832	29,629	36,930

### Identification and characterization of lncRNAs

The clean reads were mapped to the *M. truncatula* genome (Mt4.0) using the TopHat [39]. Transcripts were then assembled and annotated using the Cufflinks package [40]. Known mRNAs were identified according to the latest annotation of the *M. truncatula* genome sequence, and this led to the identification of 31,034, 36,482, 29,770, 36,832, 29,629 and 36,930 unique mRNAs from the six cDNA libraries, respectively (Table 2). The remaining reads were filtered according to length and coding potentials, such that transcripts smaller than 200 bp were excluded and transcripts with the coding potentials greater than –1 were removed. The remaining transcripts were considered as putative lncRNAs.

**Fig. 1:**
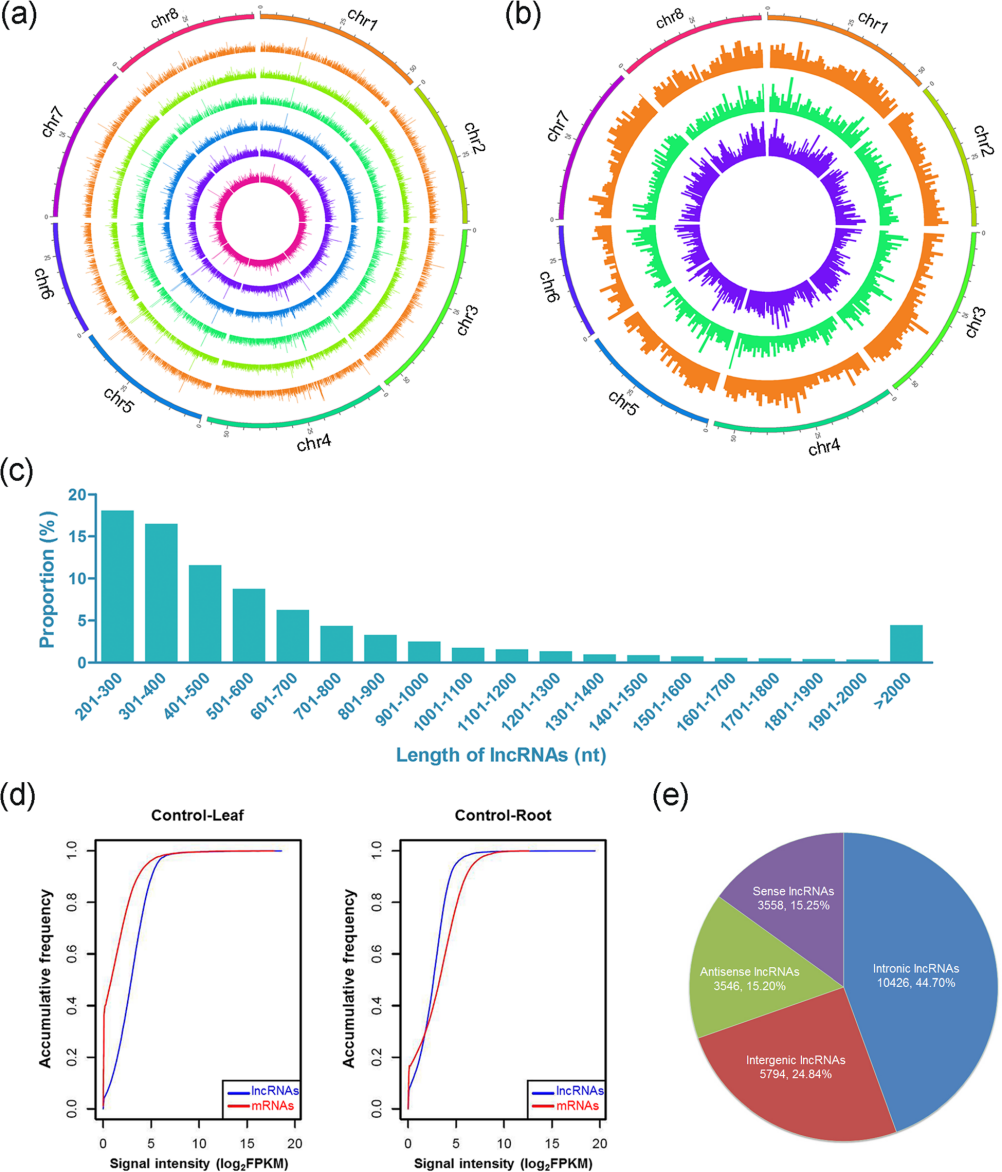
Characteristics of *M. truncatula* lncRNAs. **a** The expression level of lncRNAs (log_10_FPKM) along the eight *M. truncatula* chromosomes. It comprises six concentric rings, and each corresponds to a different sample. They are control in leaves (CK-L), control in roots (CK-R), osmotic stress in leaves (OS-L), osmotic stress in roots (OS-R), salt stress in leaves (SS-L) and salt stress in roots (SS-R) from outer to inner, respectively. **b** Distribution of different types of lncRNAs. The intronic, intergenic and sense/antisense lncRNAs are represented by different concentric rings from outer to inner, according to the loci of lncRNAs in the genome. **c** Length distribution of lncRNAs. **d** Accumulative frequency of lncRNAs and mRNAs in two control samples. Data from other samples is shown in Additional file 1: Figure S2. **e** Composition of different types of lncRNAs

**Fig. 2:**
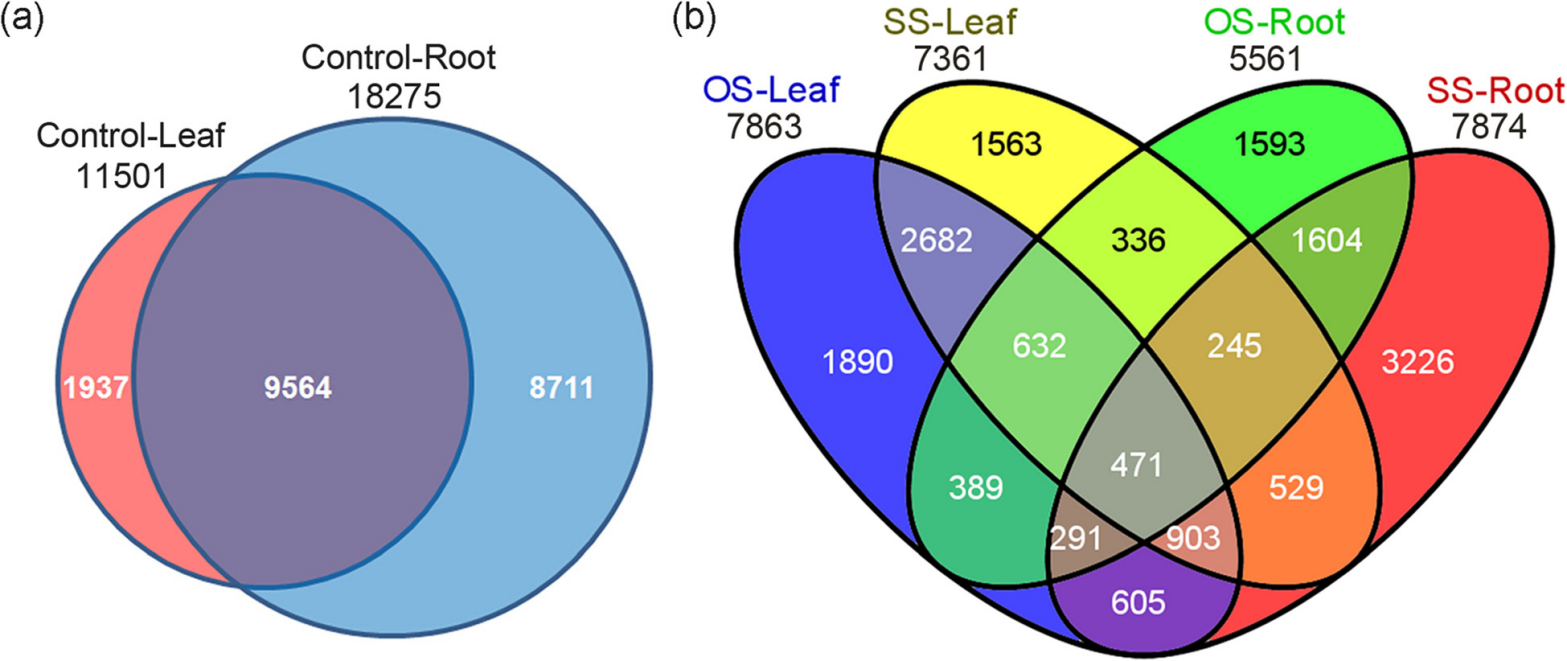
Venn diagram of common and specific lncRNAs. **a** The number of common/specific lncRNAs identified in leaves and roots under non-stressed, control conditions. **b** The number of common/specific lncRNAs between osmotic stress-responsive and salt stress-responsive lncRNAs

From these analyses, we identified 11,501, 18,275, 8,571, 18,277, 10,458 and 19,186 unique lncRNAs from the six cDNA libraries, respectively (Table 2). In total, 23,324 unique lncRNAs were obtained in the present study (Additional file 2: Table S1). And this number was similar to that of lncRNAs in *Arabidopsis* and maize [30, 41]. We found that these lncRNAs were more evenly distributed across the 8 chromosomes in *M. truncatula* with no obvious preferences of locations (Fig. 1a). According to the locations of lncRNAs in the genome, 10,426 intronic, 5,794 intergenic, 3,558 sense and 3,546 antisense lncRNAs were identified (Fig. 1b and e). In terms of the lncRNAs’ length, the majority of lncRNAs was relatively short. For example, 84.1 % of them were shorter than 1,000 nt (Fig. 1c). Interestingly, lncRNAs and mRNAs were much more abundant in roots than in leaves, given that similar amounts of raw reads were obtained for both leaf and root samples. In all libraries, more lncRNAs were detected in roots than in leaves (Table 2). For example, 18,275 lncRNAs were identified in roots, while there were 11,501 lncRNAs in leaves under control condition (Fig. 2a). Furthermore, we found that the accumulative frequency of lncRNAs differed in leaves from that in roots. The proportion of lncRNAs with a high level of expression was more than mRNAs in leaves, but this expression pattern was in contrary in roots under the control conditions (Fig. 1d). Moreover, these patterns of expression were not altered by treatments with osmotic and salt stress (Additional file 1: Figure S2). The lack of chloroplast-derived RNAs in roots might be a possible reason for the difference between leaves and roots.

All putative lncRNAs in *M. truncatula* were aligned with lncRNAs in *A. thaliana* from NONCODE database [42]. We can only detect 140 lncRNAs that were comparable to those lncRNAs in *A. thaliana*, suggesting that lncRNAs are weakly conserved between the two species (Additional file 2: Table S1). Moreover, lncRNAs which were from transposons or which encoded microRNAs were marked (Additional file 2: Table S1).

### Responses of lncRNAs to osmotic and salt stresses

To identify osmotic stress- and salt stress-responsive lncRNAs, the normalized expression (fragments per kilobase of exon per million fragments mapped, FPKM) of lncRNAs was compared amongst the six libraries.

**Fig. 3:**
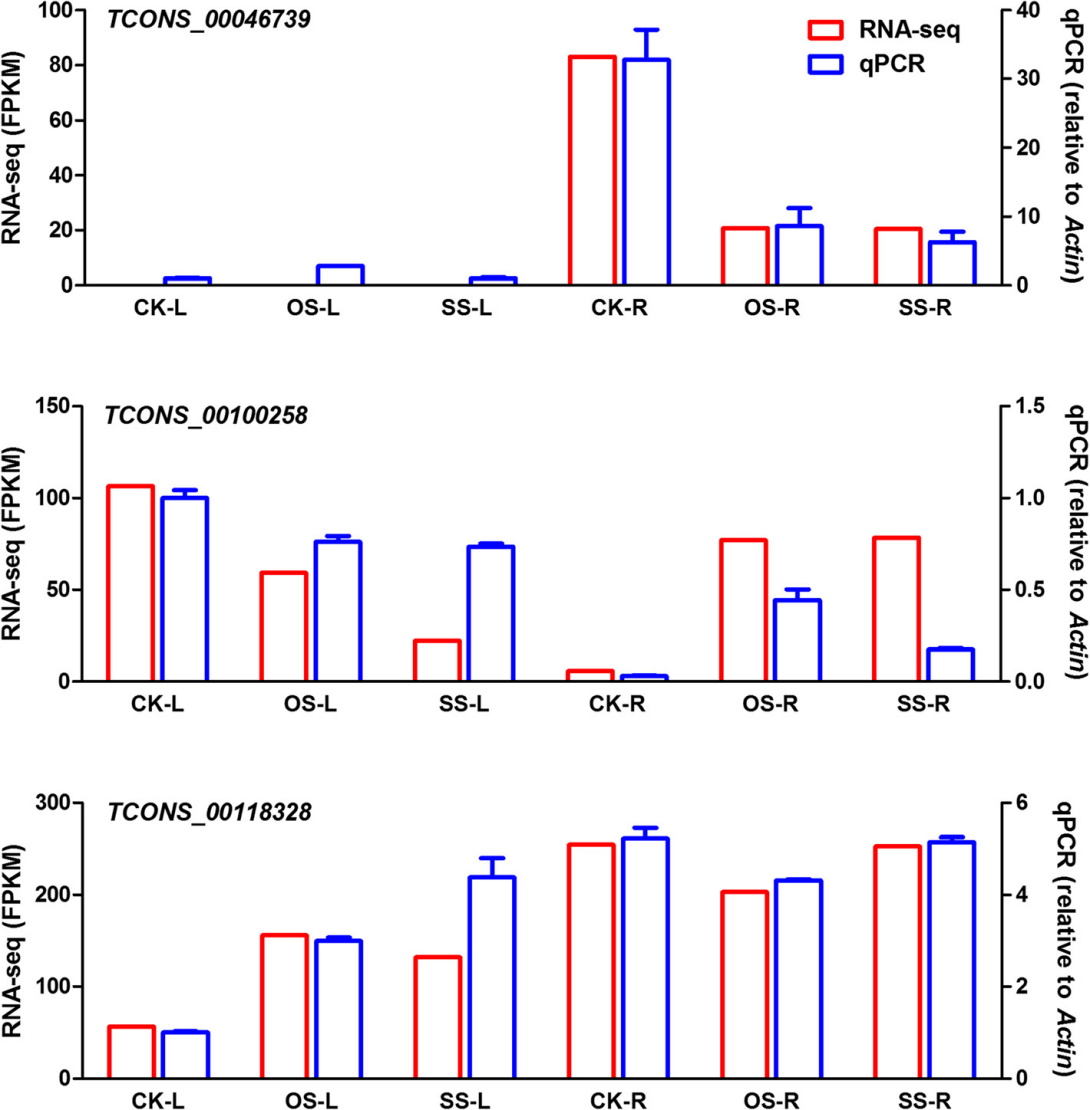
Compare of expressional results between RNA-seq and qRT-PCR. The results of three lncRNAs are shown here. Data of all 12 lncRNAs are shown in Additional file 1: Figure S3

LncRNAs that were responsive to osmotic and salt stresses in leaves and roots were identified by determining the *P*-value and false discovery rate. To verify the results from the RNA-seq experiments, 12 lncRNAs were selected to verify their expression by quantitative real-time PCR (qRT-PCR) (Fig. 3 and Additional file 1: Figure S3). These results indicate that our transcriptomic analysis is highly reproducible and reliable, and that lncRNAs identified from the high throughput sequencing represent real transcripts.

Transcript levels of 7,863 lncRNAs in leaves and 5,561 lncRNAs in roots were detected to be changed by the osmotic stress, and 7,361 lncRNAs in leaves and 7,874 lncRNAs in roots were identified to be responsive to the salt stress. Venn diagrams showed common and specific lncRNAs, whose expression was altered in roots and leaves by osmotic and salt stresses (Fig. 2b). Some lncRNAs in leaves and roots showed different responses to osmotic and salt stresses. There were 1,783 and 2,148 lncRNAs, whose expression was changed in both leaves and roots by osmotic and salt stresses, respectively. In leaves, more than half of stress-responsive lncRNAs were common between osmotic stress (59.6 %) and salt stress (63.7 %). However, these values were decreased to 47.0 % and 33.2 % in roots, respectively. The expression levels of 471 lncRNAs were found to be changed in the four treated samples (Fig. 2b). Among the lncRNAs, whose expression was changed in responses to osmotic and salt stresses, we further classified them to up-regulated and down-regulated classes (Additional file 1: Figure S4). For examples, 2,236 and 2,477 lncRNAs in leaves were up-regulated in responses to osmotic and salt stresses, respectively, and 475 lncRNAs shared similar expression patterns in responses to these two stresses. Twenty-eight and 213 lncRNAs were found to be up-regulated and down-regulated, respectively, in both roots and leaves treated with osmotic and salt stresses.

### Functional analysis of stress-responsive lncRNAs

**Fig. 4:**
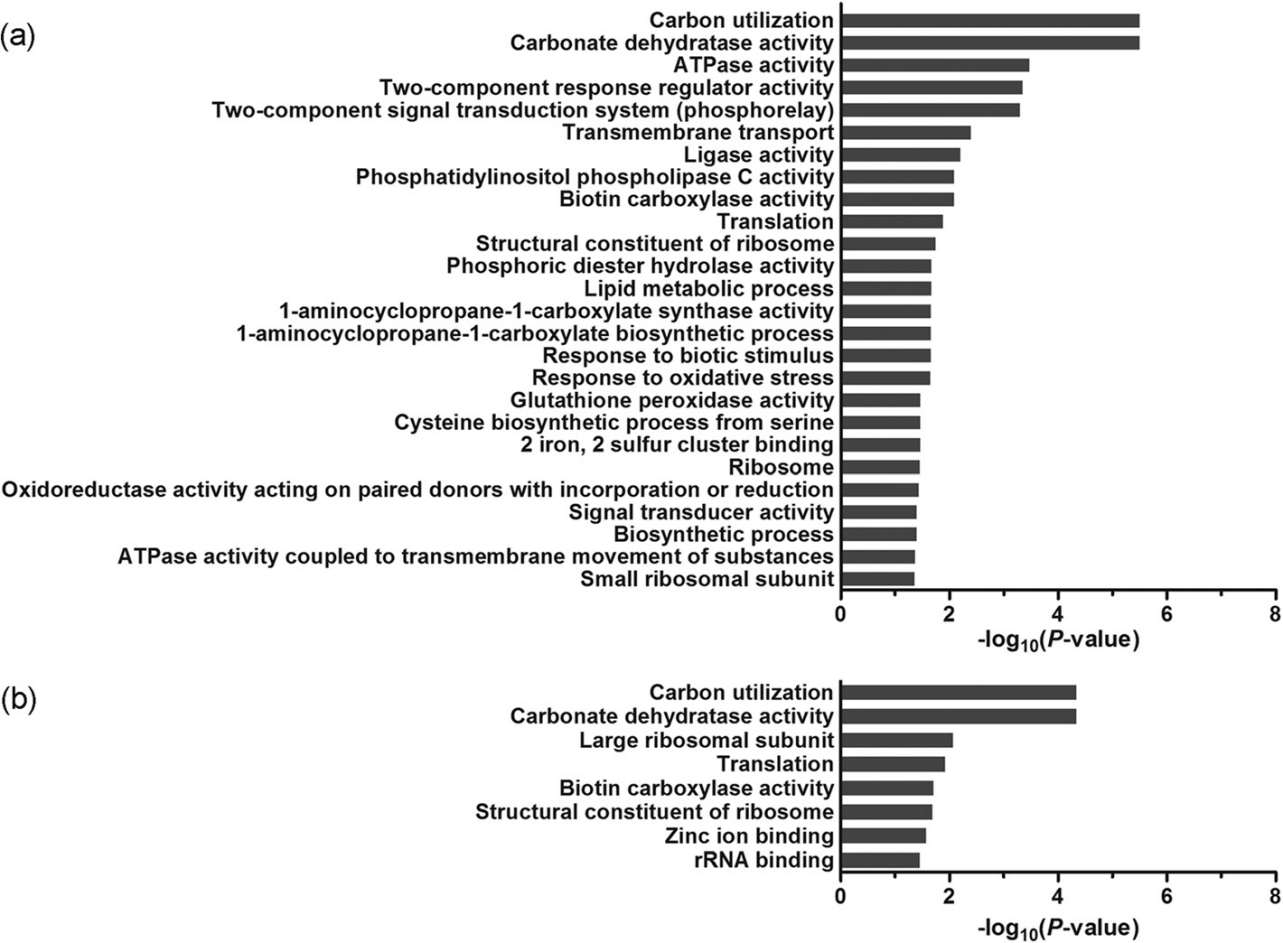
GO enhancements in leaves of *M. truncatula* under osmotic stress (**a**) and salt stress (**b**). The reliability is calculated by –log_10_ (*P*-value)

Previous studies showed that lncRNAs are preferentially located in a close proximity to genes that they regulate [13, 43–45]. To reveal potential functions of the identified lncRNAs, we analyzed Gene Ontology (GO) terms of genes that were co-expressed and spaced by less than 100 kb with the stress-responsive lncRNAs. We detected significant enrichments (*P* < 0.05) of 26 and 8 GO terms in leaves under osmotic stress and salt stress, respectively (Fig. 4, Additional file 1: Tables S2 and S3). For examples, we found GO term enrichments in cellular component (GO:0015934, large ribosomal subunit), molecular functions (GO:0004089, carbonate dehydratase activity; GO:0004075, biotin carboxylase activity; GO:0003735, structural constituent of ribosome; GO:0008270, zinc ion binding; GO:0019843, rRNA binding) and biological processes (GO:0015976, carbon utilization; GO:0006412, translation). In roots, GO term enrichments were greater than those in leaves (i.e., 52 vs 37), suggesting that roots are more sensitive to osmotic and salt stresses than leaves (Additional file 1: Figure S5, Tables S4 and S5). These findings suggest that the stress-responsive lncRNAs may regulate genes involved in many biological processes, including signal transduction, energy synthesis, molecule metabolism, detoxification, transcription and translation in response to osmotic and salt stresses.

**Fig. 5:**
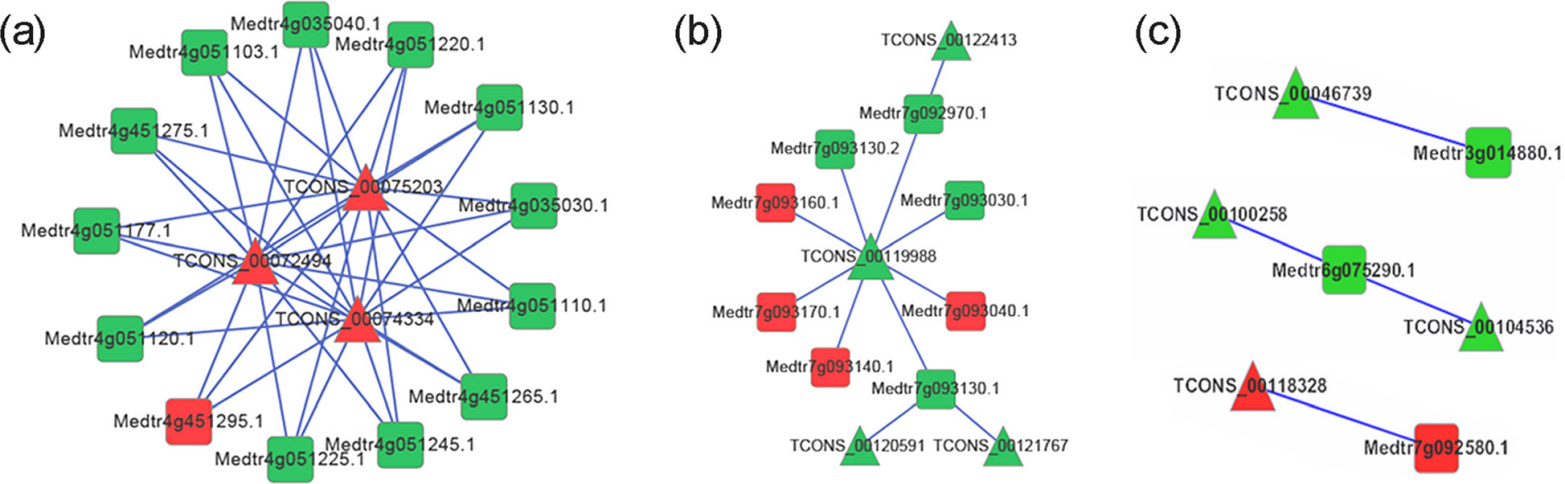
Representatives of predicted interaction networks among lncRNAs and protein-coding RNAs. The triangular and foursquare nodes represent lncRNAs and protein-coding genes, respectively. The up-regulated and down-regulated nodes are colored in red and green, respectively. Edges depict regulatory interactions among nodes

One lncRNA may regulate multiple other lncRNAs and protein-coding genes, and *vice versa* [4]. To unravel the relationship among lncRNAs and protein-coding RNAs which were co-expressed and spaced by less than 100 kb, putative interactive networks were constructed using Cytoscape (Fig. 5 and Additional file 1: Figure S6). About half of them had less than or equal to three nodes like networks in Fig. 5c. More complex interactive networks were also observed. For example, thirteen protein-coding genes involved in oxidation/reduction reaction, transcription, energy synthesis and signal transduction were found to be regulated by three lncRNAs in the situation of salt stress in leaves (Fig. 5a). Two transcription factors of MYB and zinc finger families were found in the network of Fig. 5b, which may activate stress-responsive genes in the downstream under osmotic stress in roots. The expression of lncRNAs in Fig. 5c has been validated in Fig. 3. *TCONS_00046739* was identified as regulator of cytochrome P450 in roots under salt stress. The targets of *TCONS_00100258* and *TCONS_00118328* may be two transmembrane proteins in leaves under salt stress. These networks among lncRNAs and protein-coding genes may play important roles in sensing and responding to osmotic and salt stresses. The construction of putative network based on gene expression and vicinity of the lncRNAs and protein-coding genes may not be very robust due to the few number of samples used. Future studies to validate the regulatory relationships between lncRNAs and protein-coding genes by specifically investigating the functions of lncRNAs are warranted.

**Fig. 6:**
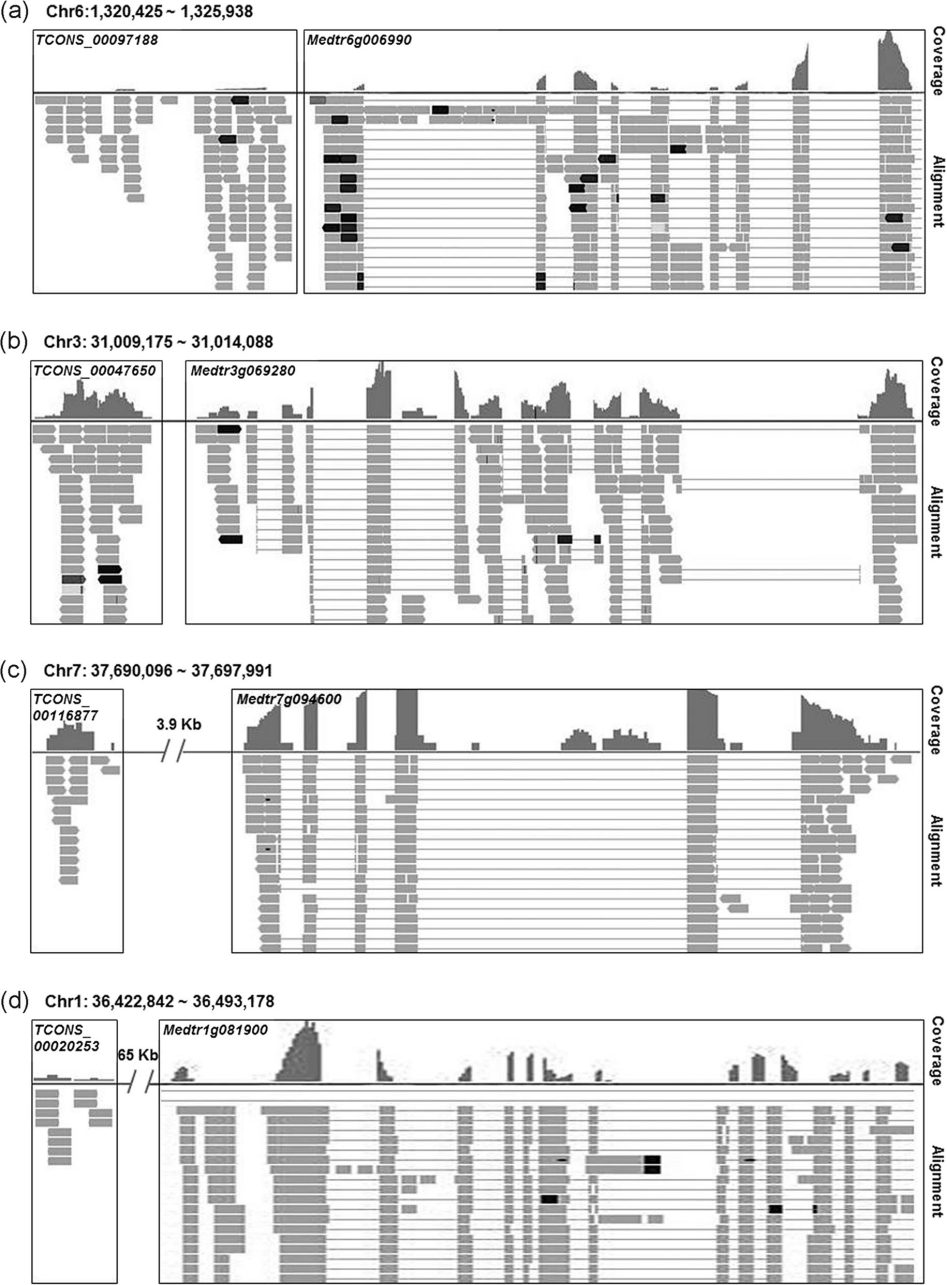
Structure of lncRNAs and their putative targets. Each figure has two separate panels showing the read coverage and alignment of RNA-Seq data. In the panel of read coverage, the height represents the expression level of corresponding loci in the genome; in alignment of RNA-Seq panel, the rectangles represent the regions which can be transcribed

Under stresses, many GO terms were enriched, such as carbonate dehydratase activity (GO:0004089) and carbon utilization (GO:0015976) that are highly significant (because of the lowest *P* value) in leaves under osmotic and salt stresses (Fig. 4). The carbonic anhydrase gene *Medtr6g006990*, belonging to these two GO terms was down-regulated by these two abiotic stresses. This gene is predicted to be regulated by the lncRNA *TCONS_00097188* located in the upstream of the coding region of *Medtr6g006990* (Fig. 6a). Carbonic anhydrase catalyzing the reversible hydration of CO_2_ into bicarbonate plays an essential role in the accumulation of CO_2_ in the active site of rubisco [46]. Our results suggest that *TCONS_00097188* may regulate photosynthesis under the abiotic stresses by regulating the expression of *Medtr6g006990*.

Under conditions of abiotic stresses, signal transduction networks are mobilized to cope with the stressed environment. The pathway of phospholipids metabolism has been proposed to be an important in response to a number of abiotic stresses [47]. For example, drought and salt stresses up-regulate the expression of genes encoding phosphatidylinositol-specific phospholipase C (PI-PLC), which hydrolyzes phosphatidylinositol 4,5-bisphosphate to the secondary messenger molecules inositol 1,4,5-trisphosphate and diacylglycerol [47]. In the present study, the expression of a PI-PLC gene (*Medtr3g069280*), which belongs to GO:0004435 (Phosphatidylinositol phospholipase C activity) and GO:0007165 (Signal transduction) was up-regulated in response to osmotic and salt stresses, and the lncRNA *TCONS_00047650* was expressed from the regulatory region of *Medtr3g069280* (Fig. 6b). These results suggested that *TCONS_00047650* may regulate the expression of *Medtr3g069280*.

Plants under osmotic and salt stresses often display oxidative stress symptoms as indicated by marked accumulation of reactive oxygen species (ROS), which damages membrane systems. To cope with the excessive accumulation of ROS, plants mobilize antioxidant enzymes to scavenge ROS [48]. We found that the expression of *Medtr7g094600* coding for glutathione peroxidase (POD) was up-regulated in roots. We identified the lncRNA *TCONS_00116877* located approximately 3.9 kb upstream of the coding sequence of *Medtr7g094600* (Fig. 6c). These results suggest that *TCONS_00116877* may be involved in regulating plant’s tolerance to the oxidative stress by modulating the expression of POD.

Effect of salinity on plant growth can be divided into ionic toxicity and osmotic stress [49]. Plants often exhibit similar tolerance mechanisms, such as altered energy synthesis, phospholipids signal transduction and detoxification to osmotic and salt stresses [47]. In addition, we found that the expression of the Na^+^/H^+^ exchanger (NHX) gene *Medtr1g081900* was up-regulated by the salt stress in roots. This gene codes for a vacuolar Na^+^/H^+^ antiporter mediating Na^+^ influx into the vacuoles [50]. This gene is predicted to be regulated by the lncRNA *TCONS_00020253* located in the upstream of the coding region of *Medtr1g081900* (Fig. 6d). These results suggest that *TCONS_00020253* is likely a regulator of *Medtr1g081900*.

## Discussion

Less than 2 % of the human genome sequences codes for proteins [51]. However, transcription is not limited to protein-coding regions [17, 52]. In fact, more than 90 % of the human genome sequences are likely transcribed [17]. These non-coding transcribed sequences are from introns, intergenic regions or the antisense strand of protein-coding genes [16]. An increasing number of studies have shown that ncRNAs play important roles in many vital biological processes, highlighting that ncRNAs are not transcriptional “noises” [4].

Studies on lncRNAs are less extensive in plants than in mammals, and those studies are mainly conducted in *A. thaliana* [25, 26]. In addition to cereals, legumes are the most important sources for human foods and animal feeds worldwide. Moreover, legumes are unique among cultivated plants for their ability to directly utilize atmospheric nitrogen through symbiotic interactions with the soil bacteria rhizobia [32]. According to the genome sequences of *M. truncatula*, only about 17 % of the sequences code for proteins [33]. Previous studies in *M. truncatula* have been concentrated upon protein-coding sequences associated with nodulation, abiotic stresses and developmental processes [53–56]. Several recent studies have investigated functions of small RNAs involved in nodulation and abiotic stresses [57–59]. In this report, we show that lncRNAs are distributed in almost the entire genome of *M. truncatula*, suggesting that lncRNA-coding regions are much more widespread than protein-coding regions (Fig. 1a and b). Whole genome sequencing and annotation facilitate functional studies of protein-coding genes [32, 33]. Identification and characterization of the large number of lncRNAs in *M. truncatula* in the present study provide valuable information for functional characterization of lncRNAs in plants in general and in legumes in particular.

In the present study, the reverse transcription was made by using complementary sequences of artificial adaptors to enrich lncRNAs with or without poly(A) tails. To distinguish sense from antisense lncRNAs, strand-specific libraries were constructed and paired-end sequencing was carried out in the present study. As a result, our results can be used to identify different types of lncRNAs to facilitate functional studies. Moreover, the abundant original data (56.7 G) generated in the present study allow us to detect lncRNAs that have low expression levels. Given that the expression of lncRNAs is highly tissue-specific [30], lncRNAs from both leaves and roots of *M. truncatula* were sequenced and their expression patterns were compared. In addition, we also sequenced and compared the expression of protein-coding genes in both leaves and roots under control and stressed conditions. This information is useful for predicting putative targets of lncRNAs. Furthermore, we identified common and specific lncRNAs from leaves and roots treated with osmotic or salt stresses to study potential functions of lncRNAs in plant’s responses to abiotic stresses. To our best knowledge, this is the first report of a comprehensive set of lncRNAs isolated from osmotic-and salt-stress treated leaf and root samples of higher plants using high-throughput sequencing. Unlike previous studies where osmotic and salt stress-responsive lincRNAs (intergenic lncRNAs) were detected in *Arabidopsis* [26] and *Populus* [60], the present study identified all types of lncRNAs involved in osmotic and salt stresses in *M. truncatula* by the strand-specific sequencing. Moreover, to make sure that the putative lncRNAs in this study conform to the criteria of length and protein-coding ability, the putative lncRNAs were selected to have >200 bp in length and less than –1 for the coding potential score. These strict criteria and improved methods made the identified lncRNAs with high sensitivity and selectivity.

To minimize the adverse effects of abiotic stresses, plants have evolved a suite of responsive mechanisms [49]. There are many protein-coding genes which are identified to play regulatory roles under varying abiotic stresses, such as *DREB1A/CBF3*, *SOS1* and so on [61–64]. However, little is known of biological functions of lncRNAs in abiotic stress responses in plants. Moreover, lncRNAs are putative potent tools for plant improvement to enhance their resistance to abiotic stresses [65]. Therefore, identification of abiotic stress-responsive lncRNAs, characterization of their functions and dissection of their regulatory networks can enhance our mechanistic understanding of plant response and adaptation to stressed environment. Several recent studies have identified lncRNAs involved in biotic/abiotic stresses in plants. *Fusarium oxysporum*, a soil-borne plant fungal pathogen, causes the vascular wilt disease through roots in several plant species [66]. LncRNAs that are responsive to *F. oxysporum* have been identified by RNA-seq, and functional characterization of these lncRNAs reveals that lncRNAs are important components of the anti-fungal networks in *A. thaliana* [66]. For abiotic stress responses, 76 lncRNAs have been identified from a full-length cDNA library of *A. thaliana* [25]. Of these, 22 lncRNAs have been shown involved in abiotic stress responses; overexpression of two identified lncRNAs renders plants more tolerance to salinity. However, because the full-length cDNA library was made from mRNAs with poly(A) tails, lncRNAs without poly(A) tails have not been identified in that study. In our present study, reverse transcription was made by complementary sequences of artificial adaptors, thus, lncRNAs with or without poly(A) tails were obtained. Liu et al. [26] identified 6,484 lincRNAs, of which 1,832 lincRNAs are responsive to drought, cold, salinity and/or abscisic acid. In a recent study, a total of 504 drought-responsive lincRNAs has been detected in *Populus* [60]. However, in these studies, only lincRNAs, rather than all types of lncRNAs, are analyzed. In our study, all types of lncRNAs, including those of sense, antisense, intronic and intergenic lncRNAs were identified using the advanced sequencing technology and analytic methods such as strand-specific sequencing and Cuffcompare analysis.

## Conclusions

In this study, we identified 23,324 putative lncRNAs from six RNA-seq libraries of *M. truncatula* by high-throughput sequencing, of which 11,641 and 13,087 lncRNAs are found to be responsive to osmotic stress and salt stress, respectively. Of these, 5,634 lncRNAs are found to be responsive to both osmotic and salt stress. We analyzed GO terms of genes that either overlap with or are immediate neighbors of the stress-responsive lncRNAs. We found enrichments of GO terms in many biological processes, including signal transduction, energy synthesis, molecule metabolism, detoxification, transcription and translation. Moreover, a number of complex interaction networks were constructed based on co-expression and genomic co-location of lncRNAs and protein-coding genes. These results suggest that lncRNAs are likely involved in regulating plant’s responses and adaptation to osmotic and salt stresses in complex regulatory networks with protein-coding genes. These findings provide valuable information for further functional characterization of lncRNAs in responses of plants in general and *M. truncatula* in particular to abiotic stresses.

## Methods

### Plant materials and stress treatments

Seeds of *Medicago truncatula* ecotypes Jemalong A17 were treated with concentrated sulfuric acid for 8 min, and then thoroughly rinsed with water. After chilled at 4 °C for 2 days, seeds were sown on 0.8 % agar to germinate at 25 °C till the radicals being about 2 cm. The seeds were planted in the plastic buckets filled with aerated nutrient solution under controlled conditions (26 °C day/22 °C night, and 14-h photoperiod). The composition of full-strength nutrient solution is: 2.5 mM KNO_3_, 0.5 mM KH_2_PO_4_, 0.25 mM CaCl_2_, 1 mM MgSO_4,_ 100 μM Fe-Na-EDTA, 30 μM H_3_BO_3_, 5 μM MnSO_4_, 1 μM ZnSO_4_, 1 μM CuSO_4_ and 0.7 μM Na_2_MoO_4_ with pH of 6.0.

Three-week-old seedlings were transferred into nutrient solutions containing either 265 mM mannitol or 150 mM NaCl, which had identical osmolality, for 5 h. Leaves and roots from at least ten individual plants were collected and frozen immediately in liquid nitrogen until use. At the same time, *M. truncatula* seedlings grown in the full-strength solution without mannitol or NaCl were harvested and were used as control. The regimes of treatment used in this study were chosen based on previous studies [25, 67].

### Construction of cDNA libraries and high-throughput sequencing

To construct libraries, total RNA was extracted from leaves and roots of seedlings grown in different solutions (osmotic stress, salt stress and control) using the Trizol (Invitrogen) according to the manufacturer’s protocols. Ribosome RNA of six RNA samples was removed using Ribo-Zero™ Magnetic Kit (Epicentre). Thereafter the strand-specific sequencing libraries were constructed following a previously described protocol [68]. The paired-end sequencing (2 × 100 bp) was performed on an Illumina Hiseq2000 sequencer at the LC Biotech, Hangzhou, China.

### Reads mapping and transcriptome assembling

The resulting directional 100 bp paired-end reads were quality-checked with FastQC (http://www.bioinformatics.babraham.ac.uk/projects/fastqc/), and adapter contaminations and low quality tags in the raw data were removed. Ribosome RNA data were also removed from the remaining data by alignment. Then, the clean reads from six-cDNA libraries were merged and mapped to the *M. truncatula* genome sequence (Mt4.0) using the spliced read aligner TopHat [39]. To construct transcriptome, the mapped reads were assembled *de novo* using Cufflinks [40]. All transcripts were required to be >200 bp in length.

### Identification of lncRNAs

The assembled transcripts were annotated using the Cuffcompare program from the Cufflinks package [40]. According to the annotation of *M. truncatula* genome sequence (Mt4.0), the known protein-coding transcripts were identified. The remaining unknown transcripts were used to screen for putative lncRNAs. The transcripts smaller than 200 bp were firstly excluded. Then, the coding potential for the remaining transcripts was calculated by the Coding Potential Calculator based on quality, completeness, and sequence similarity of the open reading frame to the proteins in the protein databases [69]. A transcript was deemed to be noncoding if the coding potentials are scored to be less than −1, which suggest that this transcript has no capacity of coding for proteins.

### Analysis of differential expression patterns

Expression levels of all transcripts, including putative lncRNAs and mRNAs, were quantified as FPKM using the Cuffdiff program from the Cufflinks package [40]. Differential gene expression was determined using DESeq with a *P*-value < 0.05 and a false discovery rate threshold of 5 % [70].

### Quantitative real-time PCR (qRT-PCR)

Total RNA was isolated using RNAiso Plus reagent (TaKaRa) and treated with RNase-free DNase I (Promega). About 0.5 μg RNA was reverse-transcribed into first-strand cDNA with PrimeScript® RT reagent Kit (TaKaRa). Quantitative real-time PCR (qRT-PCR) was performed using ABI Stepone Plus instrument. Gene-specific primers and internal control primers were listed in Additional file 1: Table S6. All qRT-PCR reactions were performed in triplicates for each cDNA sample with an annealing temperature of 57 °C and a total of 40 cycles of amplification. The relative expression levels were calculated by the comparative C_T_ method.

### Prediction of lncRNA function based on co-expression and genomic co-location

A number of investigations have indicated that one major function of lncRNAs is regulating the expression of neighboring protein-coding genes via epigenetic modification or transcriptional co-activation/repression [13, 43, 44]. The relative loci between lncRNAs and their neighbors can be exhibited using Integrative Genomics Viewer [71]. Moreover, differentially expressed lncRNAs and mRNAs were forecasted to play roles in regulation of tolerance to osmotic and salt stresses. Therefore, the genomic co-locational analysis of these lncRNAs and mRNAs was performed. We defined two genes as a co-expressed and co-located pair if they were co-expressed and spaced by less than 100 kb, according to the previously described method [72].

The neighbors of lncRNA genes were analyzed by Gene Ontology (GO) [73], and GO terms were enriched when significance (*P*) was less than 0.05 using Blast2GO [74]. Interaction networks among lncRNAs and protein-coding RNAs were constructed based on co-expression and genomic co-location using software Cytoscape [75].

### Accession number

RNA-seq data sets are available in the Sequence Read Archive database under accession number SRR1523070 (for CK-L), SRR1523071 (for CK-R), SRR1523072 (for OS-L), SRR1523075 (for OS-R), SRR1523077 (for SS-L) and SRR1523078 (for SS-R).

## Additional files

Additional file 1: Figure S1. The quality score (Q) value of RNA-seq from six samples. **Figure S2.** Accumulative frequency of lncRNAs and mRNAs from leaves and roots under osmosis and salt stress. **Figure S3.** Compare of expressional results between RNA-seq and qRT-PCR. **Figure S4.** The common and specific lncRNAs identified to be up-regulated (a) and down-regulated (b) in leaves and roots under osmosis and salt stress. **Figure S5.** GO enhancements in roots of *M. truncatula* under osmotic stress (a) and salt stress (b). The reliability is calculated by –log_10_ (*P*-value). **Figure S6.** The interaction networks among lncRNAs and protein-coding genes. **Table S2.** The GO enhancements in leaves under osmotic stress. **Table S3.** The GO enhancements in leaves under salt stress. **Table S4.** The GO enhancements in roots under osmotic stress. **Table S5.** The GO enhancements in roots under salt stress. **Table S6.** Sequences of primes using in this study. Additional file 2: Table S1. All putative lncRNAs identified in this study.

## Abbreviations

*FLC*: *FLOWERING LOCUS C*
FPKM: Fragments per kilobase of exon per million fragments mapped
GO: Gene ontology
lncRNAs: Long non-coding RNAs
miRNAs: microRNAs
ncRNAs: Non-coding RNAs
NHX: Na^+^/H^+^ exchanger
PI-PLC: Phosphatidylinositol-specific phospholipase C
POD: Peroxidase
Pro: Proline
qRT-PCR: quantitative real-time PCR
ROS: Reactive oxygen species
siRNAs: Small interfering RNAs

## References

[1] Struhl K (2007). Transcriptional noise and the fidelity of initiation by RNA polymerase II. Nat Struct Mol Biol.

[2] Ponjavic J, Ponting CP, Lunter G (2007). Functionality or transcriptional noise? Evidence for selection within long noncoding RNAs. Genome Res.

[3] Kim YJ, Zheng BL, Yu Y, Won SY, Mo BX, Chen XM (2011). The role of mediator in small and long noncoding RNA production in *Arabidopsis thaliana*. EMBO J.

[4] Wilusz JE, Sunwoo H, Spector DL (2009). Long noncoding RNAs: functional surprises from the RNA world. Gene Dev.

[5] Brosnan CA, Voinnet O (2009). The long and the short of noncoding RNAs. Curr Opin Cell Biol.

[6] Rinn JL, Chang HY (2012). Genome regulation by long noncoding RNAs. Annu Rev Biochem.

[7] Cabili MN, Trapnell C, Goff L, Koziol M, Tazon-Vega B, Regev A (2011). Integrative annotation of human large intergenic noncoding RNAs reveals global properties and specific subclasses. Gene Dev.

[8] Ponting CP, Oliver PL, Reik W (2009). Evolution and functions of long noncoding RNAs. Cell.

[9] Yamada K, Lim J, Dale JM, Chen HM, Shinn P, Palm CJ (2003). Empirical analysis of transcriptional activity in the *Arabidopsis* genome. Science.

[10] Wang XJ, Gaasterland T, Chua NH (2005). Genome-wide prediction and identification of *cis*-natural antisense transcripts in *Arabidopsis thaliana*. Genome Biol.

[11] Kornienko AE, Guenzl PM, Barlow DP, Pauler FM (2013). Gene regulation by the act of long non-coding RNA transcription. BMC Biol.

[12] Liu J, Wang H, Chua NH (2015). Long noncoding RNA transcriptome of plants. Plant Biotechnol J.

[13] Rinn JL, Kertesz M, Wang JK, Squazzo SL, Xu X, Brugmann SA (2007). Functional demarcation of active and silent chromatin domains in human *HOX* loci by noncoding RNAs. Cell.

[14] Zhao J, Sun BK, Erwin JA, Song JJ, Lee JT (2008). Polycomb proteins targeted by a short repeat RNA to the mouse X chromosome. Science.

[15] Sleutels F, Zwart R, Barlow DP (2002). The non-coding *Air* RNA is required for silencing autosomal imprinted genes. Nature.

[16] Derrien T, Johnson R, Bussotti G, Tanzer A, Djebali S, Tilgner H (2012). The GENCODE v7 catalog of human long noncoding RNAs: analysis of their gene structure, evolution, and expression. Genome Res.

[17] Birney E, Stamatoyannopoulos JA, Dutta A, Guigo R, Gingeras TR, Margulies EH (2007). Identification and analysis of functional elements in 1 % of the human genome by the ENCODE pilot project. Nature.

[18] Wierzbicki AT (2012). The role of long non-coding RNA in transcriptional gene silencing. Curr Opin Plant Biol.

[19] De Lucia F, Dean C (2011). Long non-coding RNAs and chromatin regulation. Curr Opin Plant Biol.

[20] Heo JB, Sung S (2011). Vernalization-mediated epigenetic silencing by a long intronic noncoding RNA. Science.

[21] Swiezewski S, Liu FQ, Magusin A, Dean C (2009). Cold-induced silencing by long antisense transcripts of an *Arabidopsis* Polycomb target. Nature.

[22] Franco-Zorrilla JM, Valli A, Todesco M, Mateos I, Puga MI, Rubio-Somoza I (2007). Target mimicry provides a new mechanism for regulation of microRNA activity. Nat Genet.

[23] Shin H, Shin HS, Chen R, Harrison MJ (2006). Loss of *At4* function impacts phosphate distribution between the roots and the shoots during phosphate starvation. Plant J.

[24] MacIntosh GC, Wilkerson C, Green PJ (2001). Identification and analysis of *Arabidopsis* expressed sequence tags characteristic of non-coding RNAs. Plant Physiol.

[25] Ben Amor B, Wirth S, Merchan F, Laporte P, d’Aubenton-Carafa Y, Hirsch J (2009). Novel long non-protein coding RNAs involved in *Arabidopsis* differentiation and stress responses. Genome Res.

[26] Liu J, Jung C, Xu J, Wang H, Deng SL, Bernad L (2012). Genome-wide analysis uncovers regulation of long intergenic noncoding RNAs in *Arabidopsis*. Plant Cell.

[27] Matsui A, Ishida J, Morosawa T, Mochizuki Y, Kaminuma E, Endo TA (2008). *Arabidopsis* transcriptome analysis under drought, cold, high-salinity and ABA treatment conditions using a tiling array. Plant Cell Physiol.

[28] Ding JH, Lu Q, Ouyang YD, Mao HL, Zhang PB, Yao JL (2012). A long noncoding RNA regulates photoperiod-sensitive male sterility, an essential component of hybrid rice. P Natl Acad Sci USA.

[29] Boerner S, McGinnis KM (2012). Computational identification and functional predictions of long noncoding RNA in *Zea mays*. PLoS One.

[30] Li L, Eichten SR, Shimizu R, Petsch K, Yeh CT, Wu W (2014). Genome-wide discovery and characterization of maize long non-coding RNAs. Genome Biol.

[31] Zhang W, Han ZX, Guo QL, Liu Y, Zheng YX, Wu FL (2014). Identification of maize long non-coding RNAs responsive to drought stress. PLoS One.

[32] Young ND, Debelle F, Oldroyd GED, Geurts R, Cannon SB, Udvardi MK (2011). The *Medicago* genome provides insight into the evolution of rhizobial symbioses. Nature.

[33] Branca A, Paape TD, Zhou P, Briskine R, Farmer AD, Mudge J (2011). Whole-genome nucleotide diversity, recombination, and linkage disequilibrium in the model legume *Medicago truncatula*. P Natl Acad Sci USA.

[34] Benedito VA, Torres-Jerez I, Murray JD, Andriankaja A, Allen S, Kakar K (2008). A gene expression atlas of the model legume *Medicago truncatula*. Plant J.

[35] Campalans A, Kondorosi A, Crespi M (2004). *Enod40*, a short open reading frame-containing mRNA, induces cytoplasmic localization of a nuclear RNA binding protein in *Medicago truncatula*. Plant Cell.

[36] Burleigh SH, Harrison MJ (1997). A novel gene whose expression in *Medicago truncatula* roots is suppressed in response to colonization by vesicular-arbuscular mycorrhizal (VAM) fungi and to phosphate nutrition. Plant Mol Biol.

[37] Wen J, Parker BJ, Weiller GF (2007). *In Silico* identification and characterization of mRNA-like noncoding transcripts in *Medicago truncatula*. In Silico Biol.

[38] Ewing B, Green P (1998). Base-calling of automated sequencer traces using phred. II Error probabilities Genome Res.

[39] Trapnell C, Pachter L, Salzberg SL (2009). TopHat: discovering splice junctions with RNA-Seq. Bioinformatics.

[40] Trapnell C, Williams BA, Pertea G, Mortazavi A, Kwan G, van Baren MJ (2010). Transcript assembly and quantification by RNA-Seq reveals unannotated transcripts and isoform switching during cell differentiation. Nat Biotechnol.

[41] Wang H, Chung PJ, Liu J, Jang IC, Kean MJ, Xu J (2014). Genome-wide identification of long noncoding natural antisense transcripts and their responses to light in *Arabidopsis*. Genome Res.

[42] Xie CY, Yuan J, Li H, Li M, Zhao GG, Bu DC (2014). NONCODEv4: exploring the world of long non-coding RNA genes. Nucleic Acids Res.

[43] Mercer TR, Dinger ME, Mattick JS (2009). Long non-coding RNAs: insights into functions. Nat Rev Genet.

[44] Yu WQ, Gius D, Onyango P, Muldoon-Jacobs K, Karp J, Feinberg AP (2008). Epigenetic silencing of tumour suppressor gene *p15* by its antisense RNA. Nature.

[45] Pauli A, Valen E, Lin MF, Garber M, Vastenhouw NL, Levin JZ (2012). Systematic identification of long noncoding RNAs expressed during zebrafish embryogenesis. Genome Res.

[46] Studer AJ, Gandin A, Kolbe AR, Wang L, Cousins AB, Brutnell TP (2014). A limited role for carbonic anhydrase in C_4_ photosynthesis as revealed by a *ca1ca2* double mutant in maize. Plant Physiol.

[47] Xiong LM, Schumaker KS, Zhu JK (2002). Cell signaling during cold, drought, and salt stress. Plant Cell.

[48] Mittler R (2002). Oxidative stress, antioxidants and stress tolerance. Trends Plant Sci.

[49] Zhu JK (2002). Salt and drought stress signal transduction in plants. Annu Rev Plant Biol.

[50] Yamaguchi T, Aharon GS, Sottosanto JB, Blumwald E (2005). Vacuolar Na^+^/H^+^ antiporter cation selectivity is regulated by calmodulin from within the vacuole in a Ca^2+^- and pH-dependent manner. P Natl Acad Sci USA.

[51] Collins FS, Lander ES, Rogers J, Waterston RH, Conso IHGS (2004). Finishing the euchromatic sequence of the human genome. Nature.

[52] Carninci P, Kasukawa T, Katayama S, Gough J, Frith MC, Maeda N (2005). The transcriptional landscape of the mammalian genome. Science.

[53] Wang TZ, Zhang JL, Tian QY, Zhao MG, Zhang WH (2013). A *Medicago truncatula* EF-hand family gene, *MtCaMP1*, is involved in drought and salt stress tolerance. PLoS One.

[54] de Zelicourt A, Diet A, Marion J, Laffont C, Ariel F, Moison ML (2012). Dual involvement of a *Medicago truncatula* NAC transcription factor in root abiotic stress response and symbiotic nodule senescence. Plant J.

[55] Xie C, Zhang RX, Qu YT, Miao ZY, Zhang YQ, Shen XY (2012). Overexpression of *MtCAS31* enhances drought tolerance in transgenic *Arabidopsis* by reducing stomatal density. New Phytol.

[56] Ge L, Peng J, Berbel A, Madueno F, Chen R (2014). Regulation of compound leaf development by *PHANTASTICA* in *Medicago truncatula*. Plant Physiol.

[57] Lelandais-Briere C, Naya L, Sallet E, Calenge F, Frugier F, Hartmann C (2009). Genome-wide *Medicago truncatula* small RNA analysis revealed novel microRNAs and isoforms differentially regulated in roots and nodules. Plant Cell.

[58] Wang TZ, Chen L, Zhao MG, Tian QY, Zhang WH (2011). Identification of drought-responsive microRNAs in *Medicago truncatula* by genome-wide high-throughput sequencing. BMC Genomics.

[59] Zhou ZS, Zeng HQ, Liu ZP, Yang ZM (2012). Genome-wide identification of *Medicago truncatula* microRNAs and their targets reveals their differential regulation by heavy metal. Plant Cell Environ.

[60] Shuai P, Liang D, Tang S, Zhang Z, Ye CY, Su Y (2014). Genome-wide identification and functional prediction of novel and drought-responsive lincRNAs in *Populus trichocarpa*. J Exp Bot.

[61] Liu Q, Kasuga M, Sakuma Y, Abe H, Miura S, Yamaguchi-Shinozaki K (1998). Two transcription factors, DREB1 and DREB2, with an EREBP/AP2 DNA binding domain separate two cellular signal transduction pathways in drought- and low-temperature-responsive gene expression, respectively, in *Arabidopsis*. Plant Cell.

[62] Jaglo-Ottosen KR, Gilmour SJ, Zarka DG, Schabenberger O, Thomashow MF (1998). *Arabidopsis CBF1* overexpression induces *COR* genes and enhances freezing tolerance. Science.

[63] Liu JP, Zhu JK (1998). A calcium sensor homolog required for plant salt tolerance. Science.

[64] Qiu QS, Guo Y, Dietrich MA, Schumaker KS, Zhu JK (2002). Regulation of SOS1, a plasma membrane Na^+^/H^+^ exchanger in *Arabidopsis thaliana*, by SOS2 and SOS3. P Natl Acad Sci USA.

[65] Liu R, Zhu JK (2014). Non-coding RNAs as potent tools for crop improvement. Natl Sci Rev.

[66] Zhu QH, Stephen S, Taylor J, Helliwell CA, Wang MB (2014). Long noncoding RNAs responsive to *Fusarium oxysporum* infection in *Arabidopsis thaliana*. New Phytol.

[67] Zhang LM, Liu XG, Qu XN, Yu Y, Han SP, Dou Y (2013). Early transcriptomic adaptation to Na_2_CO_3_ stress altered the expression of a quarter of the total genes in the maize genome and exhibited shared and distinctive profiles with NaCl and high pH stresses. J Integr Plant Biol.

[68] Borodina T, Adjaye J, Sultan M (2011). A strand-specific library preparation protocol for RNA sequencing. Methods Enzymol.

[69] Kong L, Zhang Y, Ye ZQ, Liu XQ, Zhao SQ, Wei L (2007). CPC: assess the protein-coding potential of transcripts using sequence features and support vector machine. Nucleic Acids Res.

[70] Anders S, Huber W (2010). Differential expression analysis for sequence count data. Genome Biol.

[71] Robinson JT, Thorvaldsdottir H, Winckler W, Guttman M, Lander ES, Getz G (2011). Integrative genomics viewer. Nat Biotechnol.

[72] Liao Q, Liu CN, Yuan XY, Kang SL, Miao RY, Xiao H (2011). Large-scale prediction of long non-coding RNA functions in a coding-non-coding gene co-expression network. Nucleic Acids Res.

[73] Ashburner M, Ball CA, Blake JA, Botstein D, Butler H, Cherry JM (2000). Gene ontology: tool for the unification of biology. Nat Genet.

[74] Conesa A, Gotz S, Garcia-Gomez JM, Terol J, Talon M, Robles M (2005). Blast2GO: a universal tool for annotation, visualization and analysis in functional genomics research. Bioinformatics.

[75] Shannon P, Markiel A, Ozier O, Baliga NS, Wang JT, Ramage D (2003). Cytoscape: a software environment for integrated models of biomolecular interaction networks. Genome Res.

