# Supplementary material for: Identification and characterization of long non-coding RNAs involved in osmotic and salt stress in Medicago truncatula using genome-wide high-throughput sequencing
Source: BMC Plant Biol. 2015 Jun 6;15:131. doi: 10.1186/s12870-015-0530-5 (PMC4457090; doi:10.1186/s12870-015-0530-5)
Supplement: Additional file 1: Figure S1. — The quality score (Q) value of RNA-seq from six samples. Figure S2. Accumulative frequency of lncRNAs and mRNAs from leaves and roots under osmosis and salt stress. Figure S3. Compare of expressional results between RNA-seq and qRT-PCR. Figure S4. The common and specific lncRNAs identified to be up-regulated (a) and down-regulated (b) in leaves and roots under osmosis and salt stress. Figure S5. GO enhancements in roots of M. truncatula under osmotic stress (a) and salt stress (b). The reliability is calculated by –log10 (P-value). Figure S6. The interaction networks among lncRNAs and protein-coding genes. Table S2. The GO enhancements in leaves under osmotic stress. Table S3. The GO enhancements in leaves under salt stress. Table S4. The GO enhancements in roots under osmotic stress. Table S5. The GO enhancements in roots under salt stress. Table S6. Sequences of primes using in this study. [file 12870_2015_530_MOESM1_ESM.pdf]

Figure S1 The quality score (Q) value of RNA-seq from six samples.

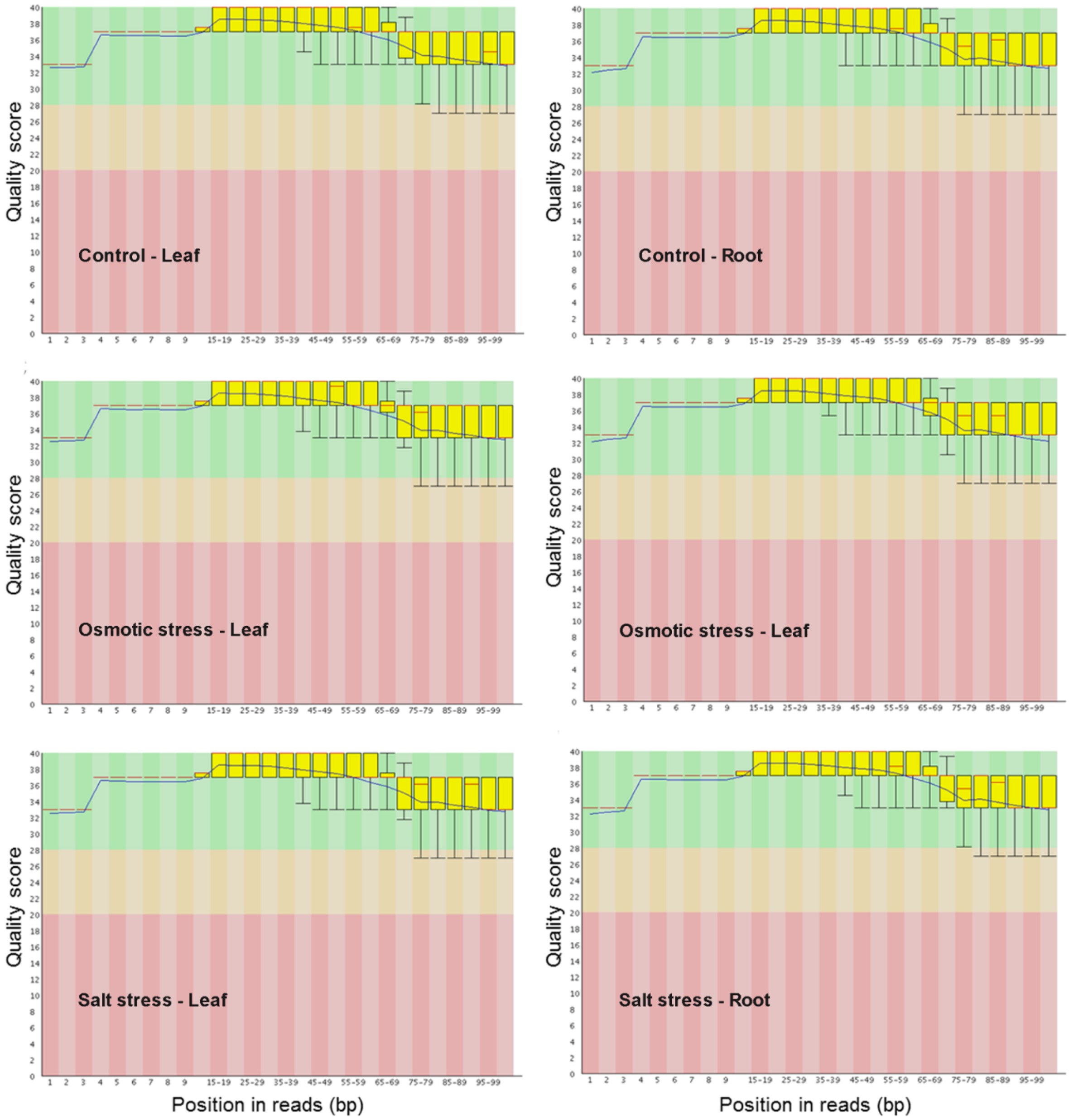

Figure S2 Accumulative frequency of lncRNAs and mRNAs from leaves and roots under osmosis and salt stress.

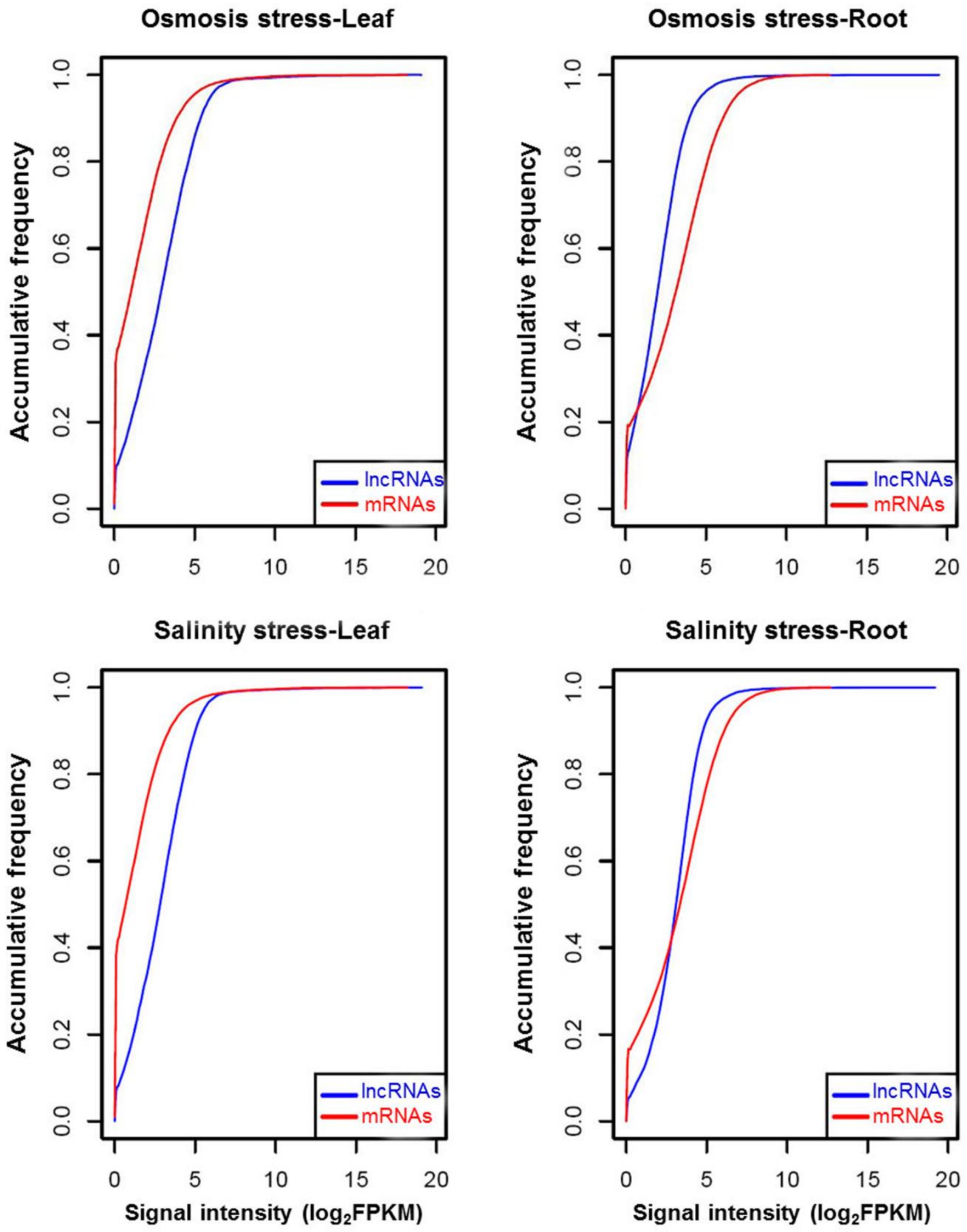

Figure S3 Compare of expressional results between RNA-seq and qRT-PCR.

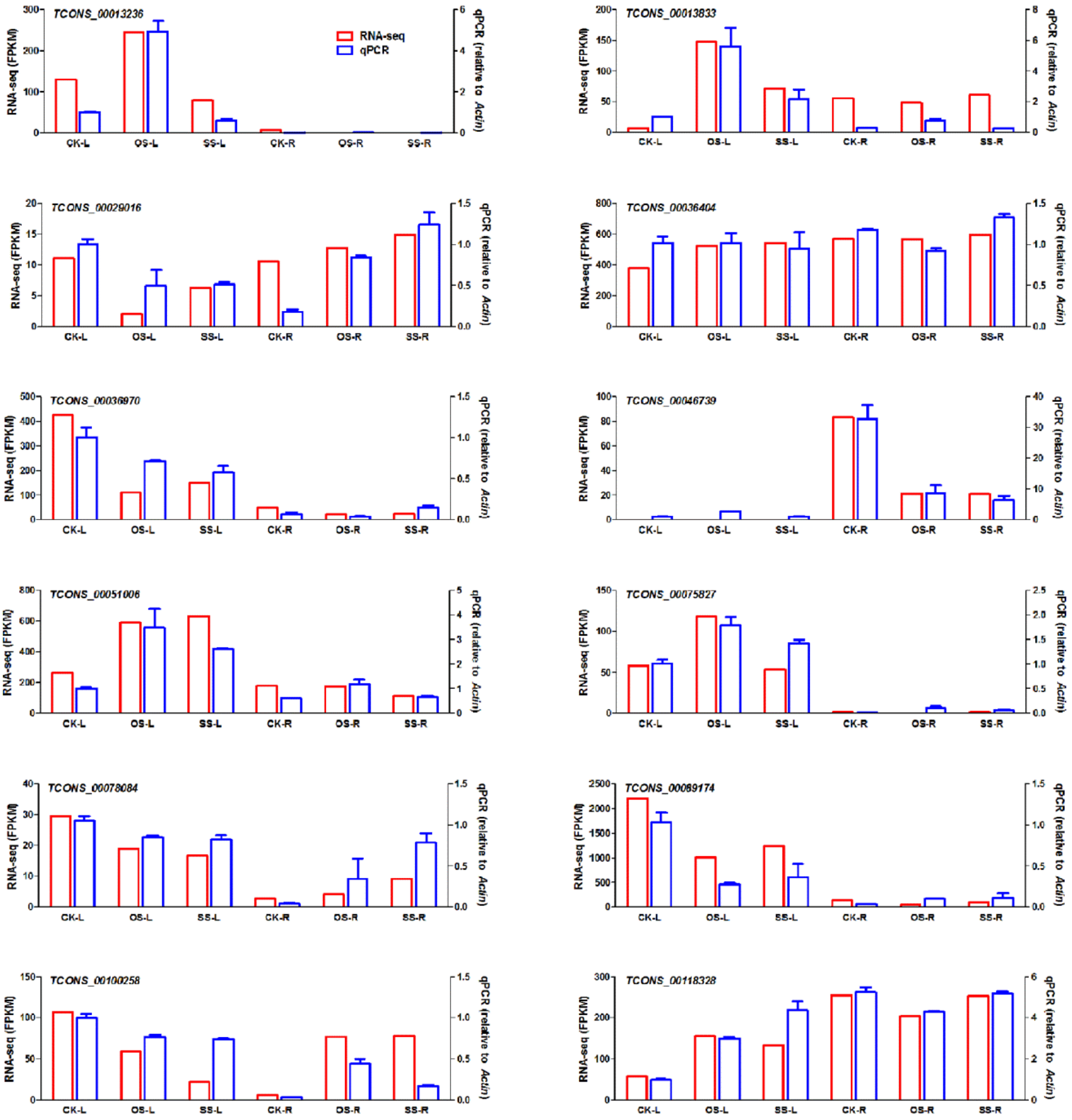

Figure S4 The common and specific lncRNAs identified to be up-regulated (a) and down-regulated (b) in leaves and roots under osmosis and salt stress.

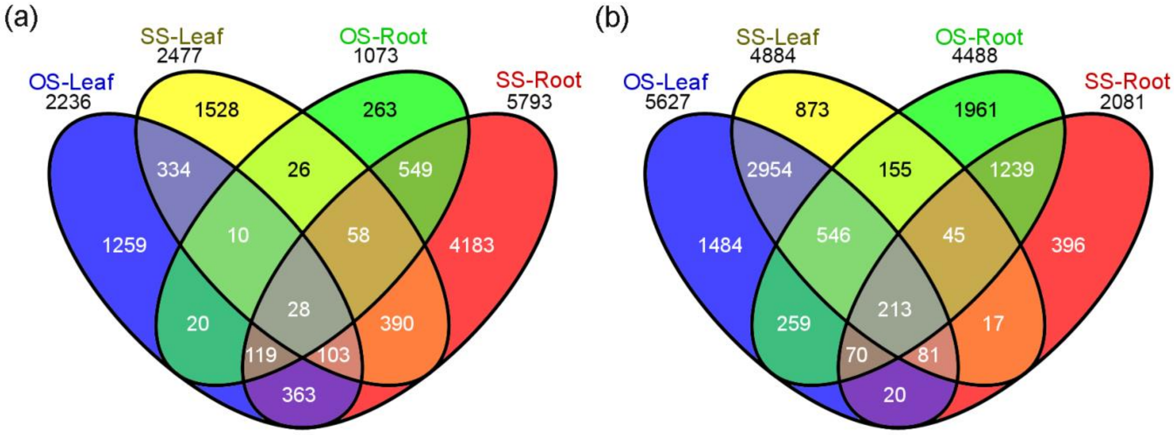

Figure S5 GO enhancements in roots of *M. truncatula* under osmotic stress (a) and salt stress (b). The reliability is calculated by $-\log_{10}$ (*P*-value).

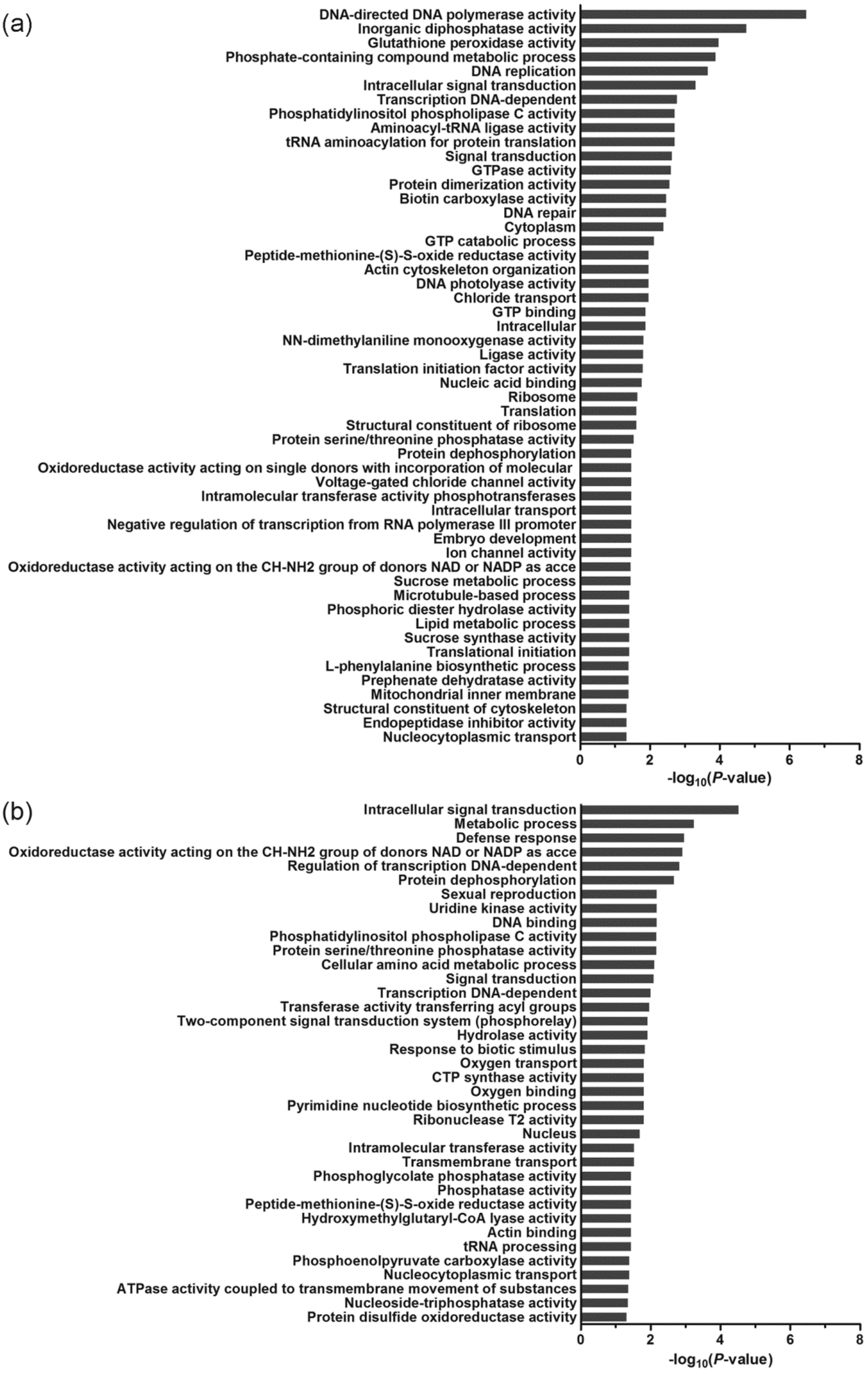

Figure S6 The interaction networks among lncRNAs and protein-coding genes.

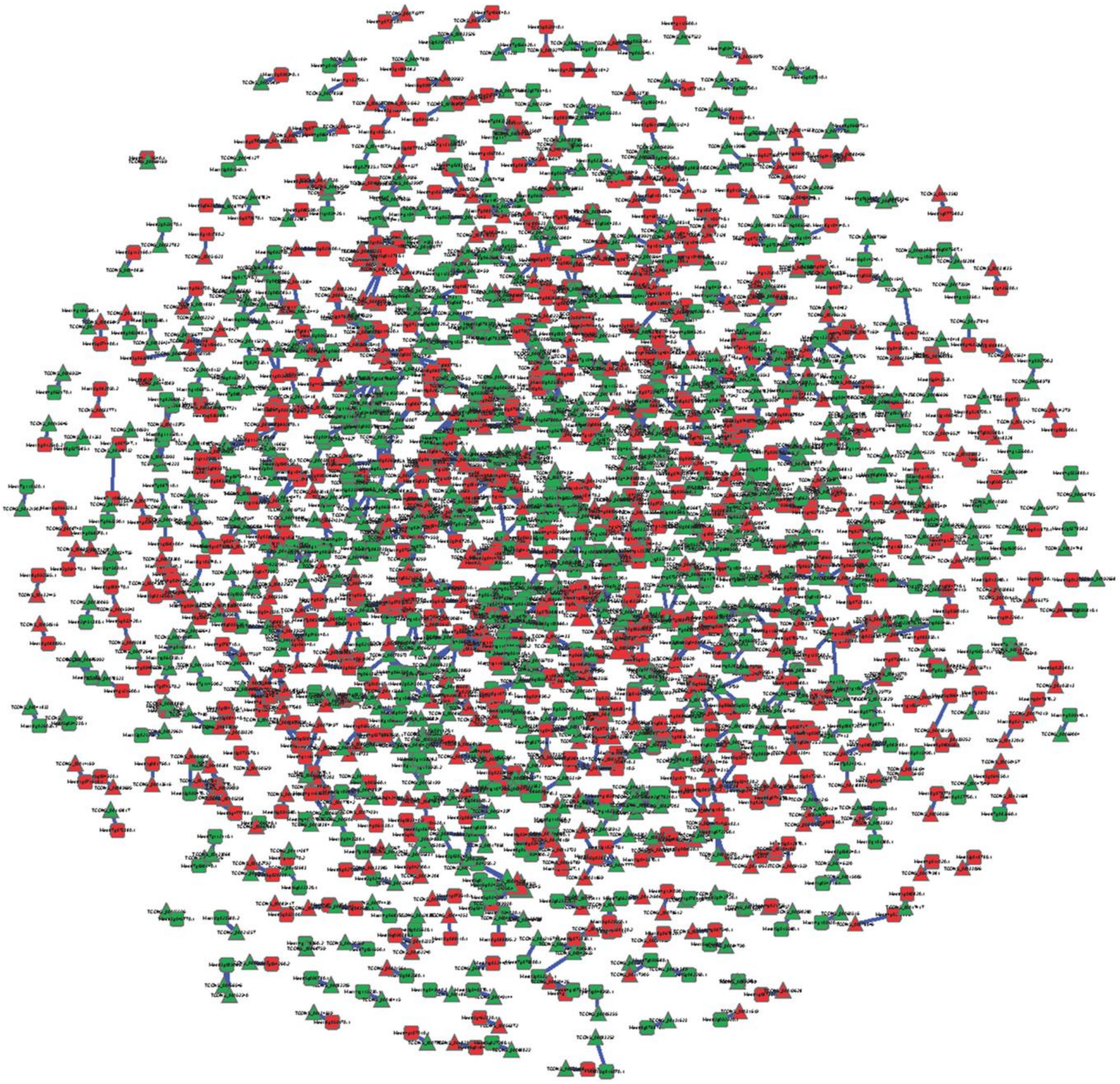

Table S2 The GO enhancements in leaves under osmotic stress

GO term	Description	$-\log_{10}(P\text{-value})$
GO:0004089	Carbonate dehydratase activity	5.53
GO:0015976	Carbon utilization	5.53
GO:0016887	ATPase activity	3.51
GO:0000156	Two-component response regulator activity	3.39
GO:0000160	Two-component signal transduction system (phosphorelay)	3.34
GO:0055085	Transmembrane transport	2.43
GO:0016874	Ligase activity	2.24
GO:0004075	Biotin carboxylase activity	2.12
GO:0004435	Phosphatidylinositol phospholipase C activity	2.12
GO:0006412	Translation	1.92
GO:0003735	Structural constituent of ribosome	1.78
GO:0006629	Lipid metabolic process	1.71
GO:0008081	Phosphoric diester hydrolase activity	1.71
GO:0009607	Response to biotic stimulus	1.7
GO:0042218	1-aminocyclopropane-1-carboxylate biosynthetic process	1.7
GO:0016847	1-aminocyclopropane-1-carboxylate synthase activity	1.7
GO:0006979	Response to oxidative stress	1.69
GO:0051537	2 iron, 2 sulfur cluster binding	1.51
GO:0006535	Cysteine biosynthetic process from serine	1.51
GO:0004602	Glutathione peroxidase activity	1.51
GO:0005840	Ribosome	1.49
GO:0016705	Oxidoreductase activity, acting on paired donors, with incorporation or reduction of molecular oxygen	1.48
GO:0009058	Biosynthetic process	1.44
GO:0004871	Signal transducer activity	1.44
GO:0042626	ATPase activity, coupled to transmembrane movement of substances	1.41
GO:0015935	Small ribosomal subunit	1.4

Table S3 The GO enhancements in leaves under salt stress

GO term	Description	$-\log_{10}(P\text{-value})$
GO:0004089	Carbonate dehydratase activity	4.38
GO:0015976	Carbon utilization	4.38
GO:0015934	Large ribosomal subunit	2.11
GO:0006412	Translation	1.96
GO:0004075	Biotin carboxylase activity	1.74
GO:0003735	Structural constituent of ribosome	1.72
GO:0008270	Zinc ion binding	1.61
GO:0019843	rRNA binding	1.5

Table S4 The GO enhancements in roots under osmotic stress

GO term	Description	$-\log_{10}(P\text{-value})$
GO:0003887	DNA-directed DNA polymerase activity	6.52
GO:0004427	Inorganic diphosphatase activity	4.8
GO:0004602	Glutathione peroxidase activity	4.01
GO:0006796	Phosphate-containing compound metabolic process	3.92
GO:0006260	DNA replication	3.69
GO:0035556	Intracellular signal transduction	3.35
GO:0006351	Transcription, DNA-dependent	2.81
GO:0006418	tRNA aminoacylation for protein translation	2.74
GO:0004812	Aminoacyl-trna ligase activity	2.74
GO:0004435	Phosphatidylinositol phospholipase C activity	2.74
GO:0007165	Signal transduction	2.66
GO:0003924	Gtpase activity	2.64
GO:0046983	Protein dimerization activity	2.59
GO:0006281	DNA repair	2.5
GO:0004075	Biotin carboxylase activity	2.5
GO:0005737	Cytoplasm	2.42
GO:0006184	GTP catabolic process	2.15
GO:0006821	Chloride transport	2
GO:0003913	DNA photolyase activity	2
GO:0030036	Actin cytoskeleton organization	2
GO:0008113	Peptide-methionine-(S)-S-oxide reductase activity	2
GO:0005525	GTP binding	1.91
GO:0005622	Intracellular	1.91
GO:0004499	N,N-dimethylaniline monooxygenase activity	1.86
GO:0016874	Ligase activity	1.84
GO:0003743	Translation initiation factor activity	1.83
GO:0003676	Nucleic acid binding	1.81
GO:0005840	Ribosome	1.68
GO:0003735	Structural constituent of ribosome	1.65
GO:0006412	Translation	1.65
GO:0004722	Protein serine/threonine phosphatase activity	1.57
GO:0006470	Protein dephosphorylation	1.51
GO:0005216	Ion channel activity	1.5
GO:0009790	Embryo development	1.5
GO:0016480	Negative regulation of transcription from RNA polymerase III promoter	1.5
GO:0046907	Intracellular transport	1.5
GO:0016868	Intramolecular transferase activity, phosphotransferases	1.5
GO:0005247	Voltage-gated chloride channel activity	1.5
GO:0016701	Oxidoreductase activity, acting on single donors with incorporation of molecular oxygen	1.5

GO:0005985	Sucrose metabolic process	1.49
GO:0016639	Oxidoreductase activity, acting on the CH-NH_2 group of donors, NAD or NADP as acceptor	1.49
GO:0006629	Lipid metabolic process	1.45
GO:0008081	Phosphoric diester hydrolase activity	1.45
GO:0007017	Microtubule-based process	1.45
GO:0006413	Translational initiation	1.45
GO:0016157	Sucrose synthase activity	1.45
GO:0005743	Mitochondrial inner membrane	1.43
GO:0004664	Prephenate dehydratase activity	1.43
GO:0009094	L-phenylalanine biosynthetic process	1.43
GO:0006913	Nucleocytoplasmic transport	1.37
GO:0004866	Endopeptidase inhibitor activity	1.37
GO:0005200	Structural constituent of cytoskeleton	1.37

Table S5 The GO enhancements in roots under salt stress

GO term	Description	$-\log_{10}(P\text{-value})$
GO:0035556	Intracellular signal transduction	4.56
GO:0008152	Metabolic process	3.28
GO:0006952	Defense response	3
GO:0016639	Oxidoreductase activity, acting on the CH-NH_2 group of donors, NAD or NADP as acceptor	2.95
GO:0006355	Regulation of transcription, DNA-dependent	2.86
GO:0006470	Protein dephosphorylation	2.71
GO:0004849	Uridine kinase activity	2.21
GO:0019953	Sexual reproduction	2.21
GO:0003677	DNA binding	2.21
GO:0004435	Phosphatidylinositol phospholipase C activity	2.21
GO:0004722	Protein serine/threonine phosphatase activity	2.2
GO:0006520	Cellular amino acid metabolic process	2.14
GO:0007165	Signal transduction	2.13
GO:0006351	Transcription, DNA-dependent	2.03
GO:0016746	Transferase activity, transferring acyl groups	2
GO:0000160	Two-component signal transduction system (phosphorelay)	1.95
GO:0016787	Hydrolase activity	1.95
GO:0009607	Response to biotic stimulus	1.87
GO:0033897	Ribonuclease T2 activity	1.84
GO:0006221	Pyrimidine nucleotide biosynthetic process	1.84
GO:0019825	Oxygen binding	1.84
GO:0003883	CTP synthase activity	1.84
GO:0015671	Oxygen transport	1.84
GO:0005634	Nucleus	1.73
GO:0016866	Intramolecular transferase activity	1.57
GO:0055085	Transmembrane transport	1.56
GO:0008033	tRNA processing	1.48
GO:0003779	Actin binding	1.48
GO:0004419	Hydroxymethylglutaryl-coa lyase activity	1.48
GO:0008113	Peptide-methionine-(S)-S-oxide reductase activity	1.48
GO:0016791	Phosphatase activity	1.48
GO:0008967	Phosphoglycolate phosphatase activity	1.48
GO:0006913	Nucleocytoplasmic transport	1.43
GO:0008964	Phosphoenolpyruvate carboxylase activity	1.43
GO:0042626	ATPase activity, coupled to transmembrane movement of substances	1.4
GO:0017111	Nucleoside-triphosphatase activity	1.39
GO:0015035	Protein disulfide oxidoreductase activity	1.35

Table S6 Sequences of primes using in this study

LncRNAs/Actin	Sense primes (5’-3’)	Antisense primes (5’-3’)
TCONS_00013236	ACAGGCCCTCAATAGAA	CGAGGAGCTTCAGAAAGA
TCONS_00013833	TCAATGTGGTCGTAGCGT	CTTGCGATGAGTTGGGTT
TCONS_00029016	TCTTCCCATGACAATACA	AATAGCCAAAGCTCTAAC
TCONS_00036404	GAGCCCTGCTTTACCTTT	GTGTCGCTAATGCGTTGC
TCONS_00036970	ATTTCATAGGCTGTGGAT	TTTACTCGGTCTGTCTCC
TCONS_00046739	CAACTCCTCCTGCAATCA	TCCAAACCTAAGACCCTA
TCONS_00051006	TCCACCACTTGGGTAGAC	TACCTGGATGGGGTCAAT
TCONS_00075827	CAGTATCAATTTAGGTGGGC	CAAATGACCGAAGTAGCG
TCONS_00078084	AGAGTGTAGCCCTGAACG	TCACTGCCGAAAGAGGAG
TCONS_00089174	TTGAGGTCGGGCAAGTAT	TGGATTTGAACCAGCGTA
TCONS_00100258	CCTTACTGGTGCTTTGTC	TTCATACCTGTTTCTCCC
TCONS_00118328	TCCGAGATTTAACCTTTAC	CTATGTTTGGGTATCTGTAT
Actin	ACGAGCGTTTCAGATG	ACCTCCGATCCAGACA
